# Effect of tendon vibration during wide-pulse neuromuscular electrical stimulation (NMES) on the decline and recovery of muscle force

**DOI:** 10.1186/s12883-017-0862-x

**Published:** 2017-05-02

**Authors:** Vanesa Bochkezanian, Robert U. Newton, Gabriel S. Trajano, Amilton Vieira, Timothy S. Pulverenti, Anthony J. Blazevich

**Affiliations:** 1 Exercise Medicine Research Institute, Edith Cowan University, Perth, Australia; 2 Centre for Sports and Exercise Science, School of Medical and Health Sciences, Edith Cowan University, Joondalup 270 Joondalup Drive, Joondalup, WA 6027 Australia; 3 UQ Centre for Clinical Research, University of Queensland, Brisbane, Australia; 4 School of Exercise and Nutrition Sciences, Queensland University of Technology, Brisbane, Australia; 5 UDF-University Centre, Brasilia, Brazil

**Keywords:** Muscle stimulation, Muscle strength, Muscle function, Muscle fatigue, Muscle damage, Meuro-rehabilitation

## Abstract

**Background:**

Neuromuscular electrical stimulation (NMES) is commonly used to activate skeletal muscles and reverse muscle atrophy in clinical populations. Clinical recommendations for NMES suggest the use of short pulse widths (100–200 μs) and low-to-moderate pulse frequencies (30–50 Hz). However, this type of NMES causes rapid muscle fatigue due to the (non-physiological) high stimulation intensities and non-orderly recruitment of motor units. The use of both wide pulse widths (1000 μs) and tendon vibration might optimize motor unit activation through spinal reflex pathways and thus delay the onset of muscle fatigue, increasing muscle force and mass. Thus, the objective of this study was to examine the acute effects of patellar tendon vibration superimposed onto wide-pulse width (1000 μs) knee extensor electrical stimulation (NMES, 30 Hz) on peak muscle force, total impulse before “muscle fatigue”, and the post-exercise recovery of muscle function.

**Methods:**

Tendon vibration (Vib), NMES (STIM) or NMES superimposed onto vibration (STIM + Vib) were applied in separate sessions to 16 healthy adults. Total torque-time integral (TTI), maximal voluntary contraction torque (MVIC) and indirect measures of muscle damage were tested before, immediately after, 1 h and 48 h after each stimulus.

**Results:**

TTI increased (145.0 ± 127.7%) in STIM only for “positive responders” to the tendon vibration (8/16 subjects), but decreased in “negative responders” (−43.5 ± 25.7%). MVIC (−8.7%) and rectus femoris electromyography (RF EMG) (−16.7%) decreased after STIM (group effect) for at least 1 h, but not after STIM + Vib. No changes were detected in indirect markers of muscle damage in any condition.

**Conclusions:**

Tendon vibration superimposed onto wide-pulse width NMES increased TTI only in 8 of 16 subjects, but reduced voluntary force loss (fatigue) ubiquitously. Negative responders to tendon vibration may derive greater benefit from wide-pulse width NMES alone.

## Background

Muscular strength is a major predictor of mortality in clinical populations, and this appears to be partly explicable by the quantity (i.e. absolute muscle volume) and quality (i.e. muscle/intra-muscular adipose content) of limb muscle mass [1, 2]. Improvements in muscle mass are also observed to be beneficial for functional mobility and quality of life as well as preventing functional decline, cardiovascular disease and hospitalization [3]. Strength training is commonly used to stimulate gains in muscle strength and has been proven to enhance longevity and quality of life in a variety of clinical populations [4–7]. However, strength training poses an increasing challenge for people with a neurological condition, such as people with spinal cord injury (SCI) who have limited ability, or are unable, to voluntarily activate their muscles.

Due to this limitation, neuromuscular electrical stimulation (NMES) has been conventionally used in clinical practice, particularly in the form of functional electrical stimulation (FES), i.e. a continuous, prolonged stimulation at low-to-moderate frequencies (30–50 Hz) paired simultaneously or intermittently with a functional task (e.g. cycling) [8]. FES exercise has been shown to slow muscle weakening or even increase muscle strength as well as reduce the rate of skeletal muscle atrophy and weakness and improve physical health in people with a SCI [9–12]. However, such interventions evoke only low relative muscle forces [9, 13] and therefore may not optimally stimulate neuromuscular strength and mass increases [14]. Instead the imposition of a higher load on the muscle with intermittent rest periods to allow continuous higher force output would be preferable [15].

Possible reasons for the lack of clinical use of high-intensity, intermittent NMES protocols include a lack of scientific exploration of its efficacy and long term functional effect [16], and their propensity to elicit rapid muscle fatigue and (possibly) muscle damage [11, 12, 17, 18]. Whilst, muscle damaging effects can be reduced with repeated exposures (i.e. repeated bout effect), the rapid muscle fatigue induced by NMES is an ongoing issue [18–21]. This rapid fatigue partly results from the use of short-pulse widths (< 300 μs), which activate muscle fibres largely through depolarization of motor axons, and which typically leads to a random motor unit recruitment pattern and therefore a substantial recruitment of fast-fatiguing type II motor units. Fatigue may also result the use of non-physiologically high stimulation frequencies (e.g. ≥ 80 Hz) [19, 22–25] and the simultaneous activation of the same motor units in repeated contractions [24, 26–28]. Therefore, the intensity and duration of stimulation that can be applied to muscles in an exercise session are reduced, thus limiting the potential for muscle force capacity, muscle mass and musculoskeletal function adaptations [29–32].

To overcome some of these problems, wide-pulse width NMES (i.e. ≥ 1000 μs) appears to be a promising tool for use in clinical populations as it appears to recruit motor units through central (i.e. indirect) pathways [33–38]. Wide-pulse width NMES can elicit asynchronous motor unit activation through the reflexive recruitment of motor neurones [39], identified by the presence of spinal H-reflexes, or asynchronous motor unit activation through the triggering of persistent inward currents (PIC) via repetitive activation of Ia afferents or post-activation potentiation of neurotransmitter release [39–41]. However, the contribution of wide-pulse width NMES to asynchronous motor unit activation through central recruitment is shown mostly during higher frequencies of stimulation (>80 Hz) and appears to have minimal effect at lower frequencies (i.e. ≤ 20 Hz), which may preclude its use in a clinical context [42]. Also, when used at higher stimulation intensities the contribution of H-reflexes to muscle activation may be minimized by orthodromic-antidromic collision, and indeed recent evidence has indicated that wide-pulse width NMES may exacerbate muscle fatigue at the higher frequencies of stimulation that may be required to elicit higher levels of muscle force [43, 44]. Thus, there is a need to consider different strategies of activating motor units in a more physiological manner for future clinical applications.

One promising method is the application of tendon vibration during muscle stimulation. Tendon vibration evokes a tonic vibration reflex through both spinal and supraspinal pathways via repetitive activation of Ia afferent fibres and possibly triggers the development of persistent inward currents at the motor neuron level [45–47]. Tendon vibration could amplify and prolong synaptic input and create a sustained depolarization leading to an increased physiological recruitment of motor units, and thus increasing muscle force output [48]. Since tendon vibration can excite only low-threshold motor units (fatigue-resistant) [49], an additional excitation of the fatigue-resistant motor units may be elicited if it is coupled with wide-pulse width NMES, and thus may result in an additional increase in muscle force output [47, 50–52]. Moreover, in some functionally important muscles, such as the quadriceps femoris, the use of wide-pulse width NMES alone may not be effective in recruiting motor units through “central pathways” [39], so the addition of tendon vibration might help to recruit Ia afferent fibres and thus increase muscle force production. Such a phenomenon has already been demonstrated in healthy people in the plantar flexors [53].

Importantly, antidromic activation of motor neurons does not occur during tendon vibration [54] as it might during electrical stimulation. Therefore, superimposing tendon vibration onto wide-pulse width NMES at low-to-moderate frequencies (e.g. 30 Hz) may induce motor neuron discharge in synchrony with the stimulus. However, it is still uncertain whether the imposition of tendon vibration onto wide-pulse width NMES could increase the peak force production and reduce the rate of muscle fatigue for a given intensity of NMES. It is also not known whether tendon vibration might increase potential muscle damaging effects due to the higher evoked forces, or instead reduce the risk by eliciting a more normal physiological excitation of the motor neuron pool.

In the present study, therefore, the hypothesis was tested that the use of tendon vibration imposed during wide- pulse width NMES (1000 μs) at a low-to-moderate frequency (30 Hz) would enhance peak muscle force, impulse performed before “fatigue”, and the post-exercise recovery of muscle function when compared to wide-pulse width NMES applied without tendon vibration in healthy individuals. These individuals also provided feedback about pain and comfort levels during the NMES protocols, so that these types of interventions might be better understood before their use in clinical populations in future research studies.

## Methods

### Participants

Sixteen healthy participants (6 women, 10 men) with no neurological or musculoskeletal disorders volunteered for the study (mean ± SD, age: 28.6 ± 7.5 y; height: 165.1 ± 27.8 cm; body mass: 77.4 ± 24.5 kg; BMI: 24.1 ± 2.2 kg/m^2^). The subjects were physically active individuals who typically performed structured physical activity 2 to 4 times a week (i.e. recreationally trained). We chose to study the effects of our interventions in healthy individuals who can provide feedback regarding the pain and discomfort experienced, because such stimuli may trigger spasticity in clinical populations such as people with spinal cord injury, stroke, brain damage or other neurological disorders [55]. Prior to the study, the participants were given detailed information about the procedures and risks of participation and they all signed an informed consent document. The participants completed the Physical Activity Readiness Questionnaire (PAR-Q) to ensure safe exercise participation and refrained from vigorous exercise (48 h) and alcohol (24 h) and stimulant consumption (e.g. caffeine, energy drinks, 6 h) prior to testing. Twelve of the 16 participants were also measured at 1 h and 48 h after the intervention to assess muscle force recovery and markers of muscle damage (details on section Muscle fatigue and muscle damage). This study was approved by the University’s Human Ethics Committee.

Study design: quasi-randomised cross-over design.

Setting of the study: Edith Cowan University. Joondalup campus. Perth. Western Australia.

### Procedures

All participants attended the laboratory on four occasions at the same time of day with a minimum of 7 days between sessions. One week prior to the first experimental session, participants attended a full familiarization session where each participant received patellar tendon vibration as well as NMES with and without patellar tendon vibration, and performed maximal voluntary isometric contractions (MVIC) of the knee extensors to ensure they could tolerate the protocols. All participants tolerated the NMES and tendon vibration protocols well. The subsequent three sessions were used to complete the following three experimental protocols in a random order without replication: 1) NMES only (STIM); 2) NMES superimposed onto tendon vibration (STIM + Vib); and 3) Vibration only (Vib). All participants (*n* = 16) were tested immediately before (PRE), immediately after (POST) and a subset of participants (*n* = 12; four participants were unable to attend all follow-up testing) were also tested one hour (1H) and 48 h (48H) after each experimental session. A standardized warm-up protocol was performed at the beginning of every session, which consisted of six consecutive concentric knee extension contractions with resistance provided by the inertia of an isokinetic dynamometer (Biodex System 3 Pro Ronkonkoma, NY) and then one repetition of isometric knee extensions at 30%, 50%, 70% and 90% of perceived maximal effort before performing a series of knee extension MVICs.

In experimental sessions, three MVICs were performed at each time point (PRE, POST, 1H and 24H) separated by 1 min of rest, with a fourth completed if a difference in peak torque of ≥3% was observed between the best two attempts. The participants were seated with hip and knee joint angles of 85° and 90°, respectively (0° = full knee extension), with the thigh and trunk secured to the dynamometer chair and the knee joint was aligned with the centre of rotation of the dynamometer. Peak isometric knee extension torque was quantified during MVIC. Participants were instructed to produce a force against the dynamometer arm by extending the knee as fast and hard as possible for 3 s. Verbal encouragement and visual feedback was provided during all MVICs.

### Neuromuscular electrical stimulation (NMES) and tendon vibration protocols

Following the MVICs, and to habituate participants to the electrical stimulations, two electrical square-wave stimuli (two 1000 μs square-wave pulses separated by 5 ms) were delivered to the dominant (stronger leg) every 20 s while the stimulation current was increased from 30 to 99 mA in 10-mA increments until a plateau in the maximum peak twitch torque was observed. Subsequently, trains of NMES were delivered by a high-voltage constant-current electrical stimulator (400 V, DS7A, Digitimer Ltd., Welwyn Garden City, UK) through four self-adhesive stimulation electrodes (Axelgaard, PALS, USA) placed over the rectus femoris (RF), vastus lateralis (VL), and vastus medialis (VM). Two 5 × 10 cm electrodes were placed over RF and one 5 × 5 electrode was placed on each of the VM and VL approximately at their motor points. The electrodes were placed to elicit the greatest twitch response with a low stimulation intensity, as determined in the familiarization session, and were marked with indelible ink on the skin to ensure identical electrode placement at subsequent sessions. The NMES protocol consisted of repeated 30-Hz trains of 58 wide-pulse width (1000 μs) symmetric biphasic pulses (0.033-s inter-pulse interval). A single train duration was 2-s and the inter-train interval was 2-s (i.e. 2-s on and 2-s off). 2-s contractions were used because extensive pilot testing revealed that shorter-duration contractions (i.e. 1 s) failed to evoke a torque plateau (i.e. maximal activation) during each train of stimulation, and that longer-duration contractions (i.e. ≥3 s) tended to elicit a rapid muscular fatigue***.*** Symmetric biphasic NMES, which employed currents with balanced positive and negative phases (polarity), was used due to its superior efficacy (in contrast to monophasic) to produce tetanic contractions and its demonstrated therapeutic benefits in clinical practice [56, 57]. The intensity of NMES was chosen to elicit 20% of the best MVIC recorded during PRE measurements for each experimental session (henceforth referred to as the ‘target torque’) by delivering three single trains of the NMES protocol with increasing stimulation intensity separated by one minute. Whilst the contribution of afferent pathways to motor unit activation occurs mainly at a high frequency (i.e. >80 Hz) and at low stimulation force levels (i.e. 10% MVC) [40–42, 53], a higher force level (i.e. ≥ 20% MVIC) and low-to-moderate frequencies (20–30 Hz) (i.e. standard clinical conditions) were chosen to elicit higher forces that should stimulate significant changes in muscle force and mass in training interventions in clinical populations. Moreover, the use of 20% MVC has been previously investigated in the plantarflexors with a clear recruitment of motor units through the reflex arc during bouts of tendon vibration [47].

**Fig. 1:**
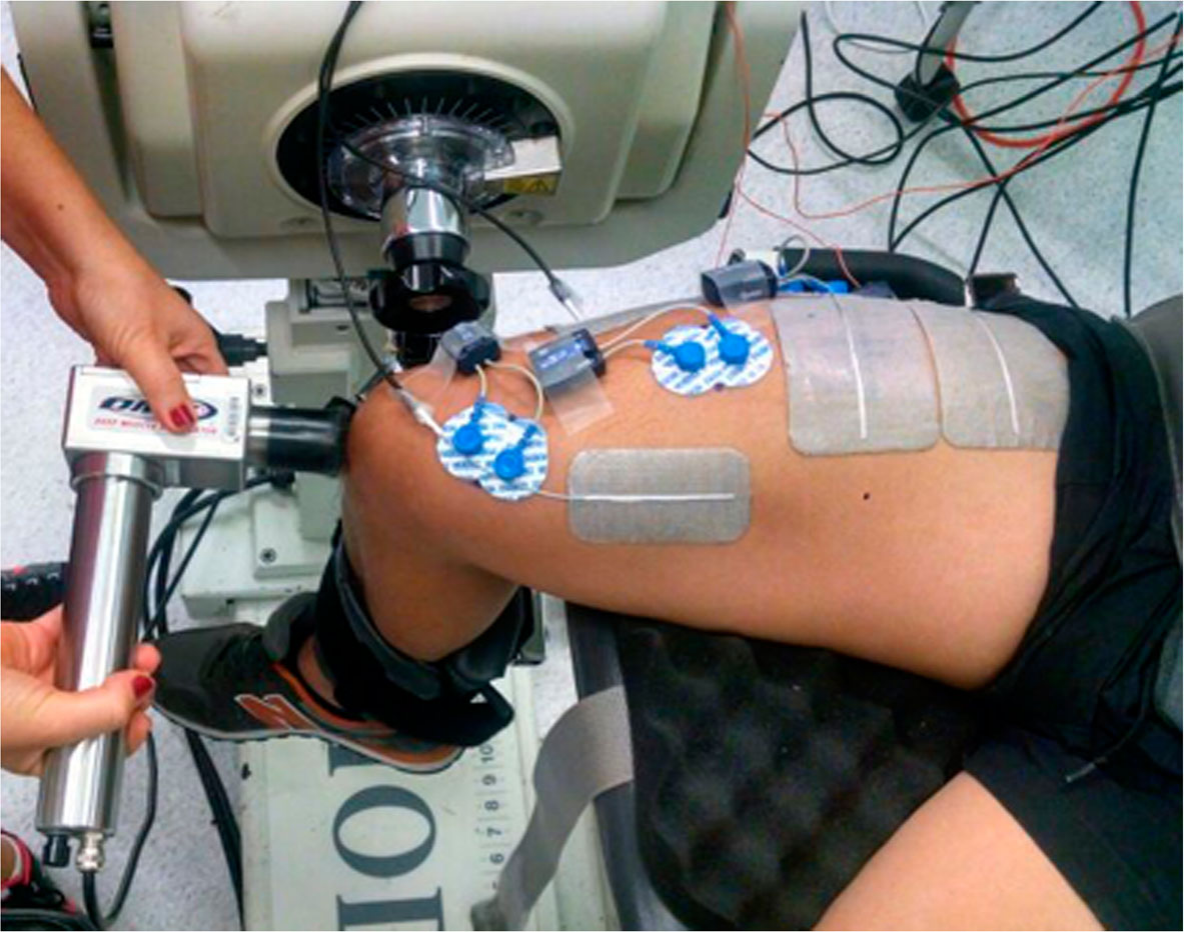
Picture showing the electrodes position on the thigh muscles and the placement of tendon vibration during one of the sessions. Patellar tendon vibration was applied with a vibration device (Deep Muscle Stimulator, Las Vegas, NV, USA) to mechanically vibrate the tendon. The tip of the vibration device was maintained at a steady pressure in a fixed position on the tendon immediately distal to the inferior border of the patella. This position was marked on the skin, and covered by a thin (1 mm thickness) soft pad to minimize pain or abrasion

Patellar tendon vibration was applied with a vibration device (Deep Muscle Stimulator, Las Vegas, NV, USA) to mechanically vibrate the tendon at 55 Hz and amplitude of 7 mm (determined by direct measurement using high-speed video capture). The tip of the vibration device was maintained at a steady pressure in a fixed position on the tendon immediately distal to the inferior border of the patella. This position was marked on the skin, and covered by a thin (1 mm thickness) soft pad to minimize pain or abrasion (refer to Fig. 1).

The three experimental interventions were:

**STIM**: electrically-evoked muscle contractions were elicited by delivering the NMES protocol until the torque was reduced to ≤60% of the target torque (i.e. 20% MVIC) in one electrically-evoked contraction, which was defined as ‘target fatigue’.

**STIM** ±**Vib**: electrically-evoked contractions delivered as in STIM, but were superimposed with patellar tendon vibration which was applied for at least 5 s before NMES and after target fatigue was reached.

**Vib:** continuous patellar tendon vibration for one minute.

### Data collection and analysis

#### Peak torque, impulse, fatigue index and number of contractions

**Fig. 2:**
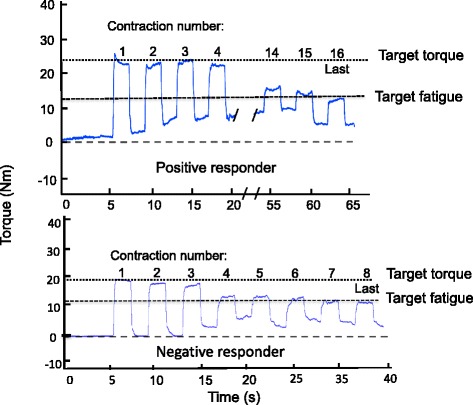
Torque production (Nm) for a positive and a negative responder during STIM + Vib. Last: last contraction before target fatigue. Target torque = 20% MVIC. Target Fatigue = 60% of target torque

The peak voluntary isometric knee extensor torque assessed during the MVIC was used to normalize the torque elicited by NMES during the sessions. Peak voluntary isometric knee extensor torque was defined as the maximum torque produced over a 500-ms window and included the plateau phase after at least a 250 ms rise time above baseline. The torque-time integral (TTI) was used to provide a measure of the total exercise stimulus received by the muscle in each condition (Fig. 2). TTI was calculated as the product of torque and time calculated from the onset of the first stimulation train (STIM) or vibration onset (STIM + Vib) to the end of the final evoked contraction at the point of target fatigue (defined on section Neuromuscular electrical stimulation (NMES) and tendon vibration protocols). Peak evoked torque was defined as the highest torque value obtained after the onset of the first stimulation train for both STIM and STIM + Vib. TTI and peak evoked torque were compared between STIM and STIM + Vib. However, some participants responded with a greater TTI after STIM + Vib (positive responders to tendon vibration) whilst others showed a lower TTI after STIM + Vib (negative responders to tendon vibration), thus a second analysis was performed after separating participants into positive and negative responder to tendon vibration groups (described on section Results). Total number of contractions was measured as the number of contractions performed from the beginning of the first evoked contraction (i.e. not including up to 3 contractions used to establish the current that elicited the target torque) reaching the target torque until the last contraction before reaching the target fatigue (defined on section Neuromuscular electrical stimulation (NMES) and tendon vibration protocols).

#### Muscle activity (EMG)

Vastus lateralis (VL), vastus medialis (VM) and rectus femoris (RF) electromyograms (EMG) were recorded using bipolar electrode configurations sampled at analogue-to-digital conversion rate of 2000-Hz (bandwidth 25–450 Hz) using a Wave wireless EMG system (Cometa Systems, Bareggio, Italy). The skin was carefully prepared by shaving, gently abrading and cleaning with alcohol prior to electrode placement. A bipolar electrode set (DE-2.1 single differential surface EMG sensor) with a 1-cm inter-electrode distance was attached to the skin over the belly of each muscle parallel to the predicted direction of muscle fibres, following the SENIAM recommendations [58]. Muscle activity was expressed as the root mean square of the EMG amplitude (applying a symmetric moving average with filter window = 500 ms) over the same time as the torque measurements, and the peak EMG was retained for analysis. Torque and EMG data were simultaneously recorded using LabChart version 8.0.2 Software (PowerLab System, ADInstruments Pty. Ltd., NSW, Australia) at the same analog-digital conversion rate.

#### Muscle fatigue and muscle damage

Muscle fatigue was determined immediately post-intervention (POST) in all participants and at 1 (1H) and 48 h (48H) in a subset of 12 participants. Muscle fatigue was calculated as the percent decrement in MVIC torque. To determine whether muscle damage may have been elicited and thus contributed to the fatigue, indirect muscle damage markers were assessed POST, 1H and 48H after the intervention. Ultrasound imaging of RF and VI muscle thickness, defined as the distance between the subcutaneous fat layer and deep muscle border were measured using B-mode axial-plane imaging (Aloka SSD-α10, Aloka Co., Ltd., Tokyo, Japan) [59]. Muscle thickness changes are considered to be an indicator of the osmotic fluid shift that results in muscle swelling subsequent to muscle damage [59]. The participants were seated on a plinth with hip and knees at 90°. The same examiner obtained images at the 50% distance between the anterior superior iliac spine and superior border of the patella. The probe was placed in a marked area in a perpendicular position using a spirit level attached to the probe. The mean of three images of the RF and VI muscle thickness measurements at the same level was obtained for each condition and time. Perception of muscle soreness was assessed using the visual analogue scale and palpation. Participants were asked to rate on a line from 0 to 100 mm (with “no pain” at 0 mm and “unbearable pain” at 100 mm) the soreness of the muscle after performing three bodyweight squats to approximately a 90° knee angle. The palpation assessment of muscle soreness consisted of the application of digital pressure using three fingers for approximately 3 s against the middle part of RF [60]. These tests have been extensively used in previous research studies evaluating indirect markers of muscle damage [60, 61].

Pain and comfort levels were also measured immediately after the completion of each NMES protocol (STIM and STIM + Vib). Subjects indicated the rate of perceived pain and comfort on a 1–10 scale based on how comfortable and painful the different protocols were perceived to be, with 1 being “comfortable and pain free” and 10 being “unbearable and extremely painful”.

### Statistical analysis

Two-way repeated measures analysis of variance (ANOVA) was used to compare changes in all variables between conditions (STIM, STIM + Vib and Vib) over time (PRE, POST, 1H and 48H) in the subset of 12 participants. A second two-way repeated measures ANOVA was used to compare STIM, STIM + Vib and Vib between PRE and POST in the full participant sample (*N* = 16). Repeated measures ANOVAs were used to compare EMG amplitude (RMS) in all individual muscles (RF, VM and VL) for STIM and STIM + Vib for PRE, POST, 1H and 48H. Pairwise *t*-tests were performed when significant interaction effects were found. Pearson’s product moment coefficients were computed to quantify the linear association between torque-time integral (TTI), peak torque and total number of contractions during STIM condition, and a binomial logistic regression analysis was performed to ascertain the ability of the torque-time integral (TTI) and total number of contractions in STIM to predict positive and negative responders to tendon vibration (i.e. TTI difference between STIM and STIM + Vib). Statistical significance was set at an alpha level of 0.05 and values were reported as mean ± SD.

We certify that all applicable institutional and governmental regulations concerning the ethical use of human volunteers were followed during the course of this research.

## Results

No significant changes were observed in any measure after Vib, thus the subsequent analysis focused on the changes in response to STIM and STIM + Vib conditions. Mean values for MVIC peak torque and surface EMG amplitudes for the Vib condition are presented in Table 1.

**Table 1:** MVIC peak torque and surface EMG amplitudes. (Mean (± SD, 95% CI)) at PRE, POST (n: 16), 1H and 48H (n: 12) for STIM, STIM + Vib and Vib conditions

Measure	PRE		POST		1H		48H	
	Mean ± SD	95% CI	Mean ± SD	95% CI	Mean ± SD	95% CI	Mean ± SD	95% CI
MVIC PT (N)								
STIM	237.8 ± 90.2	189.7–285.8	219.1 ± 90.0^*^	171.1–267.0	216 ± 72.0^*^	170.1–261.4	228.7 ± 72.2	182.8–274.6
STIM + Vib	229.3 ± 82.0	185.6–273.0	222.9 ± 84.8	177.7–268.0	222.1 ± 77.3	173.0–271.3	233.4 ± 67.2	190.6–276.0
Vib	214.2 ± 63.4	174.0–254.5	212 .8 ± 59.0	175.4–250.5	224.6 ± 73.8	177.7–271.5	230.5 ± 68.1	187.2–273.8
QUAD EMG (mV)								
STIM	1.27 ± 0.60	0.94–1.61	1.14 ± 0.48	0.88–1.40	1.20 ± 0.53	0.90–1.51	1.19 ± 0.48	0.85–1.52
STIM + Vib	1.32 ± 0.51	1.05–1.60	1.29 ± 0.52	1.01–1.56	1.38 ± 0.54	1.03–1.72	1.4 ± 0.49	1.09–1.71
Vib	1.14 ± 0.62	0.75–1.54	1.16 ± 0.60	0.78–1.54	1.16 ± 0.52	0.83–1.50	1.23 ± 0.64	0.82–1.63
RF EMG (mV)								
STIM	0.39 ± 0.16	0.30–0.48	0.32 ± 0.14 ^*^	0.25–0.40	0.35 ± 0.19	0.23–0.47	0.33 ± 0.17	0.22–0.44
STIM + Vib	0.38 ± 0.17	0.29–0.47	0.38 ± 0.19	0.28–0.49	0.39 ± 0.23	0.25–0.53	0.39 ± 0.15	0.29–0.48
Vib	0.32 ± 0.2	0.20–0.45	0.33 ± 0.19	0.21–0.45	0.33 ± 0.17	0.22–0.44	0.37 ± 0.22	0.23–0.51
VM EMG (mV)								
STIM	0.50 ± 0.44	0.27–0.74	0.48 ± 0.38	0.28–0.68	0.51 ± 0.39	0.26–0.76	0.52 ± 0.37	0.28–0.75
STIM + Vib	0.47 ± 0.33	0.29–0.64	0.46 ± 0.34	0.28–0.64	0.51 ± 0.38	0.27–0.75	0.61 ± 0.43	0.34–0.88
Vib	0.35 ± 0.34	0.14–0.57	0.35 ± 0.33	0.14–0.56	0.34 ± 0.30	0.15–0.53	0.34 ± 0.33	0.13–0.56
VL EMG (mV)								
STIM	0.38 ± 0.22	0.26–0.50	0.33 ± 0.19	0.23–0.43	0.34 ± 0.24	0.19–0.50	0.35 ± 0.30	0.16–0.54
STIM + Vib	0.48 ± 0.30	0.32–0.64	0.45 ± 0.28	0.30–0.60	0.47 ± 0.36	0.24–0.70	0.40 ± 0.27	0.23–0.57
Vib	0.46 ± 0.37	0.23–0.69	0.48 ± 0.38	0.24–0.72	0.49 ± 0.36	0.26–0.72	0.51 ± 0.42	0.25–0.78
% from PRE (baseline MVIC PT)								
STIM	−	−	-8.72 ± 5.79 ^*^	−11.80 - ^−^5.64	−6.79 ± 7.26	−11.40 - ^−^2.17	−0.67 ± 11.39	−7.91 - 6.57
STIM + Vib	−	−	−3.28 ± 6.44	−6.71 - 0.15	−1.61 ± 6.61	−5.80 - 2.59	4.52 ± 4.86	1.43–7.60
Vib	−	−	0.05 ± 5.76	−3.61- 3.71	4.42 ± 11.61	−2.95 - 11.80	7.93 ± 12.56	−0.06 -15.91

*MVI* maximal voluntary isometric contraction, *PT* peak torque, *VM* vastus medialis muscle, *VL* vastus lateralis muscle, *RF* rectus femoris muscle, *EMG*_*RMS*_ root mean square EMG amplitude, *SD* standard deviation, *95% CI* 95% Confidence Interval

^*^Significant difference from PRE (*P* <0.05)

### Torque-time integral (TTI), peak evoked torque and total number of contractions

**Fig. 3:**
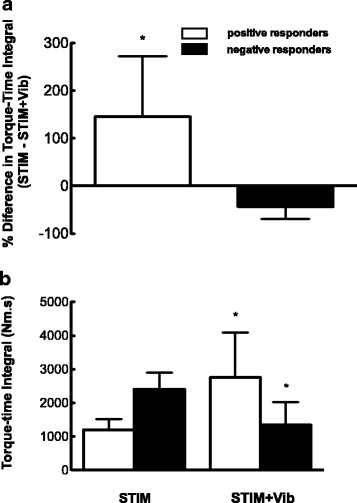
**a** Percentage difference between STIM and STIM + Vib in torque-time integral (Nm·s) for positive and negative responders (145.0 ± 127.7% and −43.5 ± 25.7%). **b** Mean torque-time integral (TTI; Nm·s) for positive responders and negative responders for STIM (1201.2 ± 321.9 Nm·s and 2402.6 ± 497.7 Nm·s) and STIM + Vib (2757.2 ± 1329.8 Nm·s and 1344.0 ± 674.6 Nm·s). *Significant difference from STIM (*P* < 0.05)

No statistical differences in peak evoked torque (*p* = 0.94), TTI (*p* = 0.56) or total number of contractions (*p* = 0.49) were observed between STIM and STIM + Vib. Nonetheless (as described in Section 2 and shown in Fig. 2) the response to STIM + Vib was clearly greater in eight participants (50% of sample) but lesser (i.e. negative) in the other eight. Thus, a positive versus negative responder to tendon vibration analysis was undertaken where positive responders to tendon vibration were defined as participants who responded with a greater TTI after STIM + Vib and negative responders to tendon vibration as participants who showed lower TTI after STIM + Vib compared to STIM (TTI: positive responders: STIM: 1201.1 ± 321.0 Nm·s; STIM + Vib: 2757.2 ± 1329.7 Nm·s; negative responders: STIM: 2402.6 ± 497.7 Nm·s; STIM + Vib: 1344.0 ± 674.6 Nm·s). This analysis revealed a group × condition interaction effect (*p* = 0.00) indicating a significant 145.0 ± 127.7% increase in TTI in STIM + Vib compared to STIM for positive responders to tendon vibration (*p* = 0.014) (see Fig. 3a), indicating an increase in the total cumulative force produced by the muscle in STIM + Vib. A significant decrease in TTI (−43.5 ± 25.7%) was observed in STIM + Vib (*p* = 0.002) in the negative responders to tendon vibration (see Fig. 3b). The mean peak evoked torques for the positive responders to tendon vibration for STIM and STIM + Vib were 49.3 ± 16.8 Nm and 52.1 ± 15.0 Nm for STIM + Vib, whilst for negative responders they were 51.7 ± 20.5 Nm and 48.1 ± 21.9 Nm. The mean total number of contractions for positive responders to tendon vibration for STIM was 16.2 ± 5.1 and for STIM + Vib was 29.1 ± 19.0, whilst the means for negative responders were 39.8 ± 25.0 for STIM and 17.1 ± 7.3 for STIM + Vib.

**Fig. 4:**
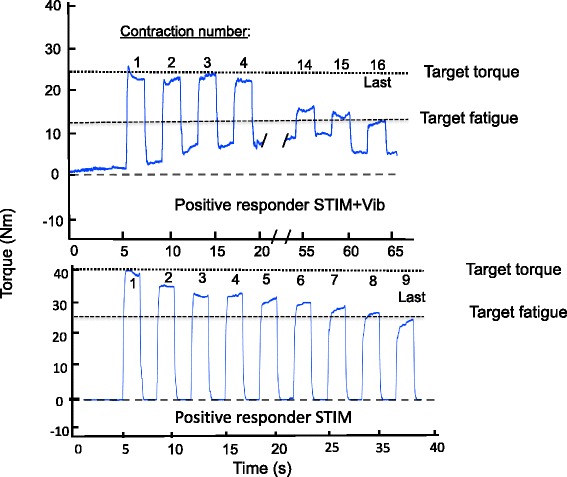
Torque production (Nm) for a positive responder during STIM + Vib and STIM. Last: last contraction before target fatigue. Target torque = 20% MVIC. Target Fatigue = 60% of target torque

Subsequent analyses of participants’ responses in STIM were undertaken to determine if the likelihood of having a positive or a negative response in STIM + Vib could be predicted. This involved examination of TTI, peak torque and total number of contractions evoked by STIM, as well as the difference in TTI between STIM and STIM + Vib. A strong and statistically significant negative correlation was observed between TTI measured in STIM (*r* = −0.72, CI 90%: -0.44 to −0.88) and the difference between the TTI measured in STIM versus STIM + Vib. Also, a correlation of −0.45 (CI 90%: -0.03 to −0.74) between TTI in STIM and the difference between STIM and STIM + Vib for the total number of contractions was observed, whilst for peak torque a correlation of −0.27 was found (CI 90%: 0.18 to −0.62). Given the strong negative relationships observed between the difference in TTI in STIM + Vib and both TTI and total number of contractions in STIM, a binomial logistic regression analysis was performed to predict the likelihood of having a positive or a negative response to STIM + Vib. The logistic regression model was statistically significant for torque-time integral (χ^2^ = 17.845, *p* < 0.0005), explaining 89% (Nagelkerke R^2^) and predicting 87.5%. For the total number of contractions (χ^2^ = 10.515, *p* < 0.0005) the model explained 64% (Nagelkerke R^2^) and predicted 81.3% of positive and negative responders to tendon vibration. Based on these results, torque-time integral and total number of contractions in STIM can be used to determine whether an individual will be a positive or negative responder to STIM + Vib in 87.5% and 81.3% of cases. Under the conditions of the present study a positive responder to tendon vibration would perform ≤16 contractions whilst a negative responder to tendon vibration would perform >16 contractions until target fatigue. An example of the response of a positive responder to tendon vibration to STIM and STIM + Vib is shown in Fig. 4.

### Peak voluntary isometric contraction (MVIC) torque

**Fig. 5:**
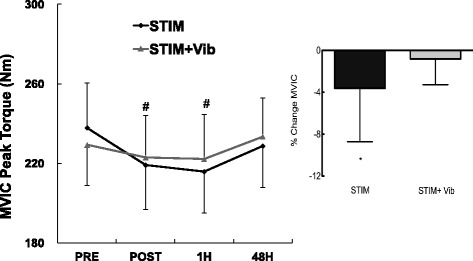
Changes in peak isometric voluntary contraction torque (MVIC) across time (PRE, POST, 1H and 48H). ^#^Significant difference from PRE (*P* < 0.05) for STIM. Mean values ± standard error (SE). *Inset*: Percentage change in MVIC from PRE to POST in STIM and STIM + Vib conditions. *Significant difference from PRE (*P* < 0.05). Mean change ± SD

As shown in Fig. 5, a time × condition interaction effect (*p* = 0.016) was observed for MVIC with a significant decrease in STIM observed from PRE (237.8 ± 90.1 Nm) to POST (219.1 ± 90.0 Nm; *p* = 0.001) and from PRE to 1H (215.8 ± 7.0 Nm; *p* = 0.007), but no change in STIM + Vib at any point. MVIC peak torques for STIM, STIM + Vib are shown in Fig. 5. The percentage change in MVIC from PRE to POST was −8.7% for STIM and −3.3% for STIM + Vib, as shown inset of Fig. 5. However, a subgroup analysis for peak voluntary isometric contraction between positive and negative responders to tendon vibration did not reveal any statistically significant difference (*p* = 0.30 for condition × time × group interaction).

### Muscle activity (EMG) during MVIC

An interaction effect (*p* = 0.006) was observed for RF EMG amplitude during MVIC, with a significant decrease (−16.7%) in the RF EMG amplitude after STIM from PRE (0.39 ± 0.16 mV) to POST (0.32 ± 0.14 mV; *p* < 0.01), but no differences for VM and VL EMG in STIM or any muscle in STIM + Vib. MVIC peak isometric torque and surface EMG amplitudes data during peak voluntary isometric torque at PRE, POST, 1H and 48H for STIM, STIM + Vib and Vib are presented in Table 1.

### Indirect markers of muscle damage: muscle thickness and muscle soreness scales; pain and comfort scale

No changes were detected in combined RF and VI muscle thickness (*p* = 0.66) or muscle soreness scales either upon palpation (*p* = 0.33) or when performing bodyweight squats (*p* = 0.37). Thus, no indications of muscle damage or soreness were observed in any condition. No statistically significant differences were found in the pain and comfort scales between STIM and STIM +Vib conditions (STIM: 4.1 ± 2.1 STIM + Vib: 5.0 ± 2.3; *p* = 0.21). Thus, both protocols elicited only “light-to-moderate” levels of pain and discomfort.

## Discussion

The main finding of the present study was that the torque-time integral (TTI) measured at the point of fatigue (i.e. 60% of initial evoked torque) was not statistically different between STIM and STIM + Vib. Based on these results, the tendon vibration superimposed onto the wide-pulse width NMES did not appear to provide any additional benefit that might not have been derived from stimulations alone under the current conditions (30 Hz, 20% MVIC in quadriceps femoris muscle). However, a significantly greater TTI was observed in a subgroup (*n* = 8) of “positive responders” to tendon vibration. Thus, in 50% of the present participants, the addition of the tendon vibration allowed for a greater total muscular work to be performed, but this was not consistent among the participants.

Another notable, and practically relevant, finding was that a significant (−8.7%) reduction in maximal voluntary torque was evoked by STIM, which persisted for at least one hour and was associated with a reduction in RF EMG amplitude; thus, the wide-pulse width NMES elicited a notable fatigue response that persisted for at least 1 h after the session and which could affect post-training movement capacity. Nonetheless, reductions in voluntary force and muscle activity were not observed when vibration was superimposed onto the NMES, even in negative responders to STIM + Vib. Therefore, the application of tendon vibration attenuated the fatigue response and allowed NMES to be used without a persisting voluntary muscle fatigue. These results are consistent with previous studies where the use of tendon vibration superimposed onto weak-to-moderate voluntary contractions was observed to attenuate the fatigue-induced decline in motor output, as assessed using standard surface EMG techniques during maximal voluntary contraction (e.G. *tibialis* anterior) [49]. Regarding the lack of effect of vibration alone (Vib) on the outcome variables, this was predictable as the vibration stimulation only recruits the lowest threshold motor units [62] and would not be sufficient to evoke strong muscle contractions, since larger, higher threshold motor units contribute more to higher force levels [63]*.*

The finding of an increased torque-time integral being produced when tendon vibration was superimposed onto NMES in the positive responders to tendon vibration (8 of 16 participants; for example see Fig. 4) shows that an increase in total muscle contractile work was achieved. This could be considered advantageous in clinical practice as it would allow the muscle to produce a greater tension for longer, and thus may better evoke chronic increases in muscle strength and mass [64, 65] and potentially improve muscle performance in people with limited voluntary muscle activation capacity (e.g. stroke, spinal cord injury, brain injury) [66]. This result in positive responders was similar to previous observations of higher force levels (up to 50% maximal voluntary contraction) elicited by tendon vibration applied simultaneously with electrical stimulation in healthy participants [53]. These greater muscular forces might possibly be attributed to the development of persistent inward currents, which could amplify and prolong the synaptic input and generate a sustained depolarization of α-motor neurons leading to an increased recruitment of fatigue resistant motor units, maximizing the use of reflexive pathways and thus increasing muscle force production [35, 40, 41, 53, 67]. The augmented torque-time integral may also be attributed to the development of tonic vibration reflexes (TVR) occuring between muscle evoked contractions only when superimposed tendon vibration is applied (see Fig. 4).

Nonetheless, 8 of 16 participants (negative responders to tendon vibration) showed a decrease in their TTI when tendon vibration was superimposed onto the wide-pulse width NMES, indicating that tendon vibration may reduce the ability to produce force and decrease the total muscle contractile work during high-intensity NMES contractions, thus representing a disadvantage in this subgroup of participants. This negative response may speculatively have been caused by the stimulation of Golgi tendon organs by the low-to-moderate frequency (55 Hz) of vibration applied during the contraction [68]. Alternatively, the additional synaptic input provided by tendon vibration might have exacerbated fatigue mechanisms (e.g. ion channel function and neurotransmitter depletion), particularly for those individuals for whom the wide-pulse width NMES has already successfully recruited the lower threshold motor units.

In this case, we can infer that negative responders to tendon vibration might benefit from the sole application of (possibly wide-pulse width) NMES (STIM) based on the similar response in total amount of work (i.e. TTI) and total number of contractions performed under STIM in comparison to positive responders to tendon vibration under STIM + Vib (see Fig. 3b). So, if a lower TTI is found after STIM then the application of tendon vibration would likely improve performance to approximately equally to the “negative responders”, whilst if a higher TTI is found after STIM then tendon vibration would likely reduce the TTI to similar levels to those found in “negative responders”. Thus, it appears that negative responders to tendon vibration will show a decrease in total muscle contractile work if tendon vibration is added and in these cases tendon vibration superimposed onto wide-pulse width NMES may represent a disadvantage and thus, the use of NMES alone would be more beneficial to elicit a high muscle force production. Whether this group has derived *ben*efits from the wide-pulse width NMES as compared to standard (i.e. narrow pulse widths) remains to be explicitly investigated in future studies. The large inter-individual variability observed in our study is consistent with previous studies using wide-pulse width NMES, where substantial individual variability exists regarding its magnitude of effect [38, 44, 69, 70]. Due to this large inter-individual variability, clinicians may need to test individual responses to tendon vibration before its implementation in clinical practice.

Additionally, given that TTI and total number of contractions measured in STIM could be used to predict 87.5% and 81.3% of the positive and negative responders to tendon vibration, respectively, the measurement of TTI or the number of contractions during wide-pulse width NMES might be a clinically relevant method to predict whether a patient would benefit from superimposed tendon vibration (i.e. a positive response to tendon vibration). In that case, clinicians might determine whether to use tendon vibration on their patients based on their response to STIM alone. However, since measuring TTI in clinical practice may not be practically feasible in some cases, using total number of contractions, for example by visually counting until reaching a pre-determined torque level (representing muscle fatigue), might be used to identify patients who will benefit from additional tendon vibration.

A secondary finding of the present study was that a significant decrease in maximal voluntary force production was observed for at least 1 h in STIM but not STIM + Vib. It is important to note that these results were not influenced by the values obtained prior to the application of the electrical stimulation protocols (MVIC PRE), as these values were not statistically different (*p* = 0.19) and reliable between days (ICC = 0.95). This result showed an advantage of the superimposed tendon vibration that prevented the significant fatigue-induced decline in MVIC. Thus, in the clinical context, tendon vibration may provide a benefit of reduced voluntary muscle fatigue when compared to moderate-frequency, wide-pulse width NMES that could allow for further rehabilitation work or improved performance of activities of daily living and occupational tasks in the hours after a rehabilitation session. It is not clear from the present data how the vibration provided a fatigue-attenuation benefit. Speculatively, it may have reduced the synchrony of the motor unit activity during NMES, which may have then reduce the rate of muscle fatigue [45, 71]. This might occur if ongoing facilitation of fatigue-resistant motor units was provided due to the generation of trains of Ia afferent signals into the spinal cord, inducing an excitation of homonymous motor neurons through the development of persistent inward calcium (Ca^2+^) or sodium (Na^+^) currents (PIC) at their dendritic trees [45, 71]. Such a mechanism would evoke a tonic vibratory reflex influencing both spinal and supraspinal pathways [45, 71]. Tendon vibration-induced primary muscle spindle endings (i.e. Ia afferent activation) might also substitute for the fusimotor-driven Ia discharge and α-motor output decline that usually occurs during sustained voluntary contractions [49, 72]. This would have attenuated the muscle fatigue response observed in our study by continuing the Ia afferent activation response. Regardless of the potential mechanism, there seems to be a reversal of central drive failure when tendon vibration is superimposed onto wide-pulse width NMES, but further tests are needed to confirm this theory. However, the levels of muscle voluntary isometric fatigue observed in the present study (−8% after STIM) were somewhat smaller than the 22–30% reported by other studies [20, 73, 74]. This discrepancy may be attributed to the use of biphasic wide-pulse width NMES, the use of a lower stimulation frequency (30 vs. 75 Hz) or different duty cycle ratio (2–2 vs. 5–15 s), or that muscles were activated to only 20% of MVIC (with ‘fatigue’ being 60% of this value) in comparison to maximal tolerable levels of MVIC used by others [20, 74, 75]. Further explanation of these possibilities is required to accurately explain the differences in voluntary fatigue.

Another important finding was a reduced RF EMG amplitude observed during MVIC in STIM but not STIM + Vib, indicating that the loss of central drive to the muscle was minimized or eliminated with the application of tendon vibration. This selective decline in RF activation, but no other muscles, at POST may be attributed to the higher activity of RF during isometric knee extension at 90° of knee flexion [76] influencing the activation of the bi-articular RF over the vastii muscles (VM and VL) during STIM [77, 78] and the higher EMG fatigue experienced on RF due to its bi-articular nature [79]. It would be of interest to determine whether the decrease in EMG during MVIC and its “rescue” when tendon vibration is imposed onto NMES is observed in other skeletal muscles, or whether it is unique to RF or other biarticular muscles [79].

Of final note, no evidence for muscle damage or soreness was found in either condition at any time point, and levels of reported pain and comfort were “light-to-moderate” and not statistically different between conditions (mean for STIM = 4/10, STIM + Vib = 5/10). Therefore, the muscle stimulation and vibratory stimuli could be applied without concern for ongoing muscle fatigue or damage, and with reasonable levels of pain and comfort, at least in healthy individuals. The muscle damaging effects of electrically evoked isometric contractions have been previously attributed to the disruption of the muscle fibres and their surrounding connective tissue [74, 80–82], which causes a prolonged loss of muscle force generating capacity. The lack of damage in the present study might be explained by the fact that muscles were activated to only 20% of MVIC, whilst maximal tolerable levels of muscle contraction were evoked in previous studies [74, 80–82]. Moreover, being able to exercise regularly without ongoing soreness or force loss may have broad clinical relevance since pain can trigger life-threatening episodes in some clinical conditions such as autonomic dysreflexia in people with spinal cord injury [55]. Finally, since the levels of pain and discomfort were “light-to-moderate” and not different between the conditions, these NMES protocols can be considered safe for implementation in future clinical studies.

Limitations of this study are that our results are only pertinent under specific conditions of NMES (1000 μs, 30 Hz) at relatively higher torque levels than previously been investigated [40–42, 53]. Thus, a different response may result under different NMES conditions. Further studies using relatively higher intensities (i.e. 20% MVIC) and the same parameters of NMES as used in this study should be performed to confirm our results.

## Conclusions

Based on the present results, the imposition of tendon vibration onto moderate-frequency wide-pulse width NMES may allow for a greater amount of muscular work to be performed, and thus for a more optimum training response to be achieved, in a proportion of participants who respond positively. However, a lesser response might be elicited in those individuals who respond negatively (50% of participants in the current study) and in these cases tendon vibration superimposed onto wide-pulse width NMES may represent a disadvantage and thus, the use of NMES alone would be more beneficial to elicit a high muscle force production. Nonetheless, the use of tendon vibration superimposed onto wide-pulse width NMES appeared to minimize the voluntary fatigue experienced after the training session and might therefore allow for additional rehabilitation work to be performed or for the trained muscle groups to be more effectively used for locomotion (i.e. crutches use) and activities of daily living after the session for both positive and negative responders to tendon vibration. Finally, since muscle damage and soreness were not observed, and levels of pain and discomfort were light-to-moderate after both NMES conditions, the application of these methods appear to be sufficiently safe to be used in clinical populations, such as in people with SCI. This is important as some clinical populations may be susceptible to high levels of muscle fatigue and muscle damage or might respond negatively to painful stimuli. Nonetheless, replication of these findings in a larger sample is encouraged before this type of NMES protocol is recommended in clinical practice.

## Abbreviations

EMG: Electromyography
MVC: Maximal voluntary contraction
MVIC: Maximal voluntary isometric contraction
NMES: Neuromuscular electrical stimulation
RF: Rrectus femoris
SCI: Spinal cord injury
STIM: Neuromuscular electrical stimulation protocol
STIM + Vib: Neuromuscular electrical stimulation superimposed onto tendon vibration protocol
TTI: Total torque-time integral
Vib: Vibration only protocol
VL: Vastus lateralis
VM: Vastus medialis
